# Enthesitis related arthritis- 2 distinct clinical phenotypes?

**DOI:** 10.1186/1546-0096-9-S1-P151

**Published:** 2011-09-14

**Authors:** Corinne Fisher, John Ioannou, Debajit Sen

**Affiliations:** 1 Centre for Rheumatology, University College London Hospital, London, UK

## Background

Enthesitis related arthritis (ERA) is the subtype of juvenile idiopathic arthritis (JIA) as defined by the International League of Associations for Rheumatology (ILAR) classification (2004). Asymmetrical lower limb arthritis and enthesitis are said to be prominent early features with axial inflammation a late feature. In our cohort of patients with ERA we observed this was often not the case- with axial inflammation occurring early and the emergence of 2 distinct clinical phenotypes.

## Aims

To identify patients with ERA who developed axial disease and compare their clinical characteristics to those with peripheral disease.

## Methods

A retrospective case review was undertaken of patients with ERA attending our adolescent rheumatology clinic. Clinical characteristics of patients with confirmed axial disease were compared to those without axial disease.

## Results

68 patients (58 males, 10 females) with ERA were identified. 36 had axial disease (aERA) confirmed on MRI scan, 32 did not have axial disease (pERA). Male predominance was similar in both groups (86.1% vs 84.4%). Median time to inflammatory spinal symptoms was 2y 8m. In the aERA group, median age of onset was higher (11y 4m vs 9y 8m) as was B27 positivity (82.8% vs 48.5%). Patients had more hip arthritis (69.4% vs 53.1%) as well as sacroiliac and lumbar spine symptoms. In pERA, patients were more likely to have ankle arthritis (78.1% vs 27.8%) and enthesitis (65.6% vs 47.2%). Extra-articular manifestations occurred only in the aERA group (acute anterior uveitis 11.1%, inflammatory bowel disease 13.9%). A higher proportion of the aERA group required anti-TNF treatment (47.2% vs 25%).

## Conclusion

Patients with aERA develop disease at an older age, have a higher rate of B27 positivity, have more hip arthritis and develop extra-articular complications more commonly. Patients with pERA have more ankle arthritis and enthesitis and are less likely to require anti-TNF therapy.

Large observational studies are needed to further characterise these 2 clinical phenotypes of ERA.

